# The neuropathologic findings in a case of progressive cavitating leukoencephalopathy due to *NDUFV1* pathogenic variants

**DOI:** 10.1186/s40478-022-01445-1

**Published:** 2022-09-26

**Authors:** Nicole Becker, Aditi Sharma, Matthew Gosse, Brooke Kubat, Kyle S. Conway

**Affiliations:** 1 Department of Pathology, University of Iowa, 200 Hawkins Drive, Iowa City, IA 52242 USA; 2 Department of Neurology, University of Iowa, 200 Hawkins Drive, Iowa City, IA 52242 USA; 3 Department of Pathology, University of Michigan, 2800 Plymouth Rd., Building 35, Faculty Suite Room 36-1221-68, Ann Arbor, MI 48109-2800 USA

**Keywords:** Progressive cavitating leukoencephalopathy, Mitochondrial leukoencephalopathy, Demyelination, Complex I

## Abstract

Pathogenic variants in the *NDUFV1* gene, which codes for complex I of the mitochondrial respiratory chain, have been associated with a variety of clinical phenotypes, including a progressive cavitating leukoencephalopathy. The neuropathology of *NDUFV1*-associated leukoencephalopathy is not well-described. We present a report of a 24-year-old female with two pathogenic variants in the *NDUFV1* gene, together with antemortem skeletal muscle biopsy and postmortem neuropathologic examination. Autopsy neuropathology showed a cavitating leukoencephalopathy with extensive white matter involvement, regions of active demyelination, and sparing of the subcortical U-fibers. Muscle biopsy showed subtle but distinct histologic abnormalities by light microscopy, and ultrastructural analysis demonstrated mitochondrial abnormalities including abnormal subsarcolemmal mitochondrial accumulation, electron-dense inclusions, and enlarged mitochondria with abnormal cristae. Our report is the first comprehensive description of the neuropathology in a patient with compound heterozygous variants in the *NDUFV1* gene and progressive cavitating leukoencephalopathy. This case is evidence of pathogenicity of one *NDUFV1* variant (c.565 T > C, p.S189P), which has not been previously described as pathogenic. These findings, in combination with the ultrastructural abnormalities in the mitochondria by electron microscopy, support the mitochondrial nature of the pathology. Together, this case highlights the link between mitochondrial abnormalities and demyelinating processes in the central nervous system (CNS).

## Introduction

Isolated deficiency in complex I of the mitochondrial respiratory chain is the most common cause of mitochondrial respiratory chain dysfunction. Complex I is the largest complex in the oxidative phosphorylation chain and is composed of 45 subunits encoded by both nuclear and mitochondrial DNA. The *NDUFV1* gene codes for a 51-kD subunit of complex I, and pathogenic variants in this gene have been implicated in complex I deficiency. Patients with *NDUFV1* pathogenic variants acquire disease in an autosomal recessive pattern of inheritance. These patients can present with a variety of clinical presentations, including Leigh syndrome, mitochondrial encephalomyopathy, lactic acidosis, and stroke-like episodes (MELAS), neonatal cardiomyopathy with lactic acidosis, fatal infantile lactic acidosis, leukodystrophy with macrocephaly and hepatopathy with renal tubulopathy [4]. More recently, complex I deficiency has been associated with progressive cavitating leukoencephalopathy, a neurodegenerative disease characterized by white matter leukoencephalopathy progressing to confluent cystic changes. Progressive cavitating leukoencephalopathy has been described in patients with pathogenic variants in *NDUFV1,* as well as the nuclear genes *NDUFS1, NDUFV2,* and *NDUFA1,* which are also involved in the formation of complex I subunits [1, 2, 5, 8, 11, 13, 14]. Despite the diversity of clinical presentations and implicated genes involved in complex 1 deficiency, no distinct genotype–phenotype correlations have been established [4].

Previously described cases of cavitating disease associated with complex I deficiency have focused on the clinical and magnetic resonance imaging (MRI) findings, rather than pathologic findings [1, 2, 5, 8, 11, 13, 14]. In a subset of the cases, enzymatic deficiency in complex I was described, as identified by muscle or skin biopsy [2, 5, 8]. One case described the brain biopsy findings in a patient with deficiency in complex I due to *NDUFA1* variants, which showed evidence of demyelination [1]. No prior studies have performed complete neuropathologic examination of the brain at autopsy in patients with *NDUFV1* pathogenic variants, nor have shown ultrastructural evidence of mitochondrial abnormalities. In this report, we present the complete neuropathologic exam of the brain, spinal cord, and skeletal muscle from a young woman with multiple clinical episodes of acute neurologic deterioration, progressive cavitary lesions on MRI, and pathogenic compound heterozygous variants in the *NDUFV1* gene*.*

## Case presentation

### Clinical presentation

Our patient was a previously healthy 24-year-old female who initially presented with new onset paresthesias, numbness, and progressive symmetric weakness involving all extremities, which worsened over the span of three weeks. Physical exam findings were notable for upper motor neuron type weakness in all extremities, positive Babinski sign, and decreased sensation to light touch in the left upper extremity. MRI imaging findings at initial presentation showed a right frontal ring enhancing lesion with surrounding edema and long segment T2-weighted spinal cord signal abnormality extending from C1-T2 concerning for a demyelinating process. Comprehensive workup for CNS demyelinating diseases, systemic malignancy, and autoimmune processes was unremarkable, and no tumor cells were identified on cerebrospinal fluid (CSF) flow cytometry and cytology. Due to her lack of clinical improvement on high dose intravenous (IV) steroids, plasmapheresis and rituximab were initiated. During her admission, she developed an episode of severe lactic acidosis requiring transfer to the intensive care unit for management, with subsequent resolution with bicarbonate infusion. A broad toxic and metabolic workup for an etiology was unremarkable. She was eventually discharged with mild improvement in her weakness.

**Fig. 1:**
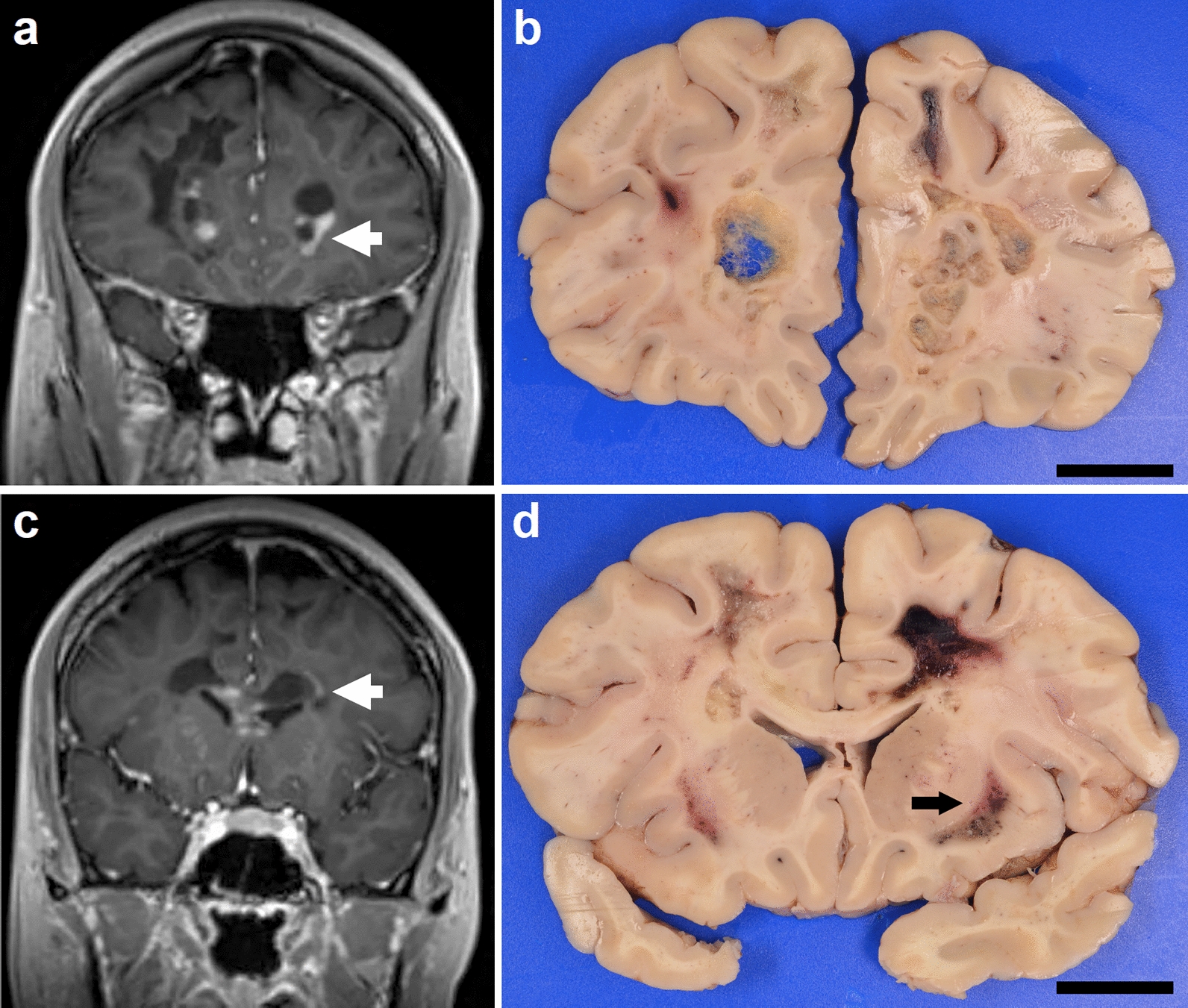
Radiologic and pathologic findings at autopsy. MRI performed several weeks before death showed cystic changes (white arrow) in the periventricular white matter with associated areas of post-contrast enhancement **a**, **c**. Gross examination at autopsy showed numerous cystic lesions in the periventricular white matter **b**, as well as solid lesions characterized by softening and dusky discoloration (black arrow) **d**. Size bar in panels b and d are 2 cm

She was readmitted 6 months later with episodes of severe lactic acidosis, worsening encephalopathy, and vomiting. Repeat imaging of the brain showed marked progression of cystic encephalomalacia involving the bilateral frontal lobes (Fig. 1a), periventricular white matter (Fig. 1c), and corpus callosum, which largely spared the grey matter. Spine imaging also showed interval progression of T2/STIR signal along the central and dorsal thoracic cord extending to the conus medullaris. At this point, workup for genetic causes of leukoencephalopathy and mitochondrial disease was initiated, which included performing a biopsy of the quadriceps muscle to evaluate for mitochondrial disease. Despite high dose IV steroids and plasmapheresis, she continued to deteriorate with worsening bouts of lactic acidosis as high as 20 mEq/L, requiring initiation of continuous renal replacement therapy. She had an episode of transient right gaze deviation and unresponsiveness with a negative workup for vascular causes and seizures. Her admission was complicated by sepsis, pneumonia causing acute hypoxic respiratory failure, cardiovascular instability requiring prolonged high dose pressors, and a possible coagulopathy. A brain biopsy of the right frontal lobe lesion was performed, whichdemonstrated clusters of foamy macrophages with loss of myelin and retention of axons. Due to worsening multi organ failure and poor neurological exam, the family elected to proceed with comfort care measures two weeks after hospital admission.

### Pathologic findings

An antemortem blood sample was sent to GeneDx (Gaithersburg, MD) for clinical whole exome sequencing and mitochondrial genome analysis, but did not result until after the patient’s death. This testing revealed compound heterozygous variants in the *NDUFV1* gene (c.365C > T, p.P122L and c.565 T > C, p.S189P). Genetic testing of the parents showed each harbored one of the variants, demonstrating that the patient’s variants were in trans. In silico analysis predicted a deleterious effect on protein and function for both variants.

**Fig. 2:**
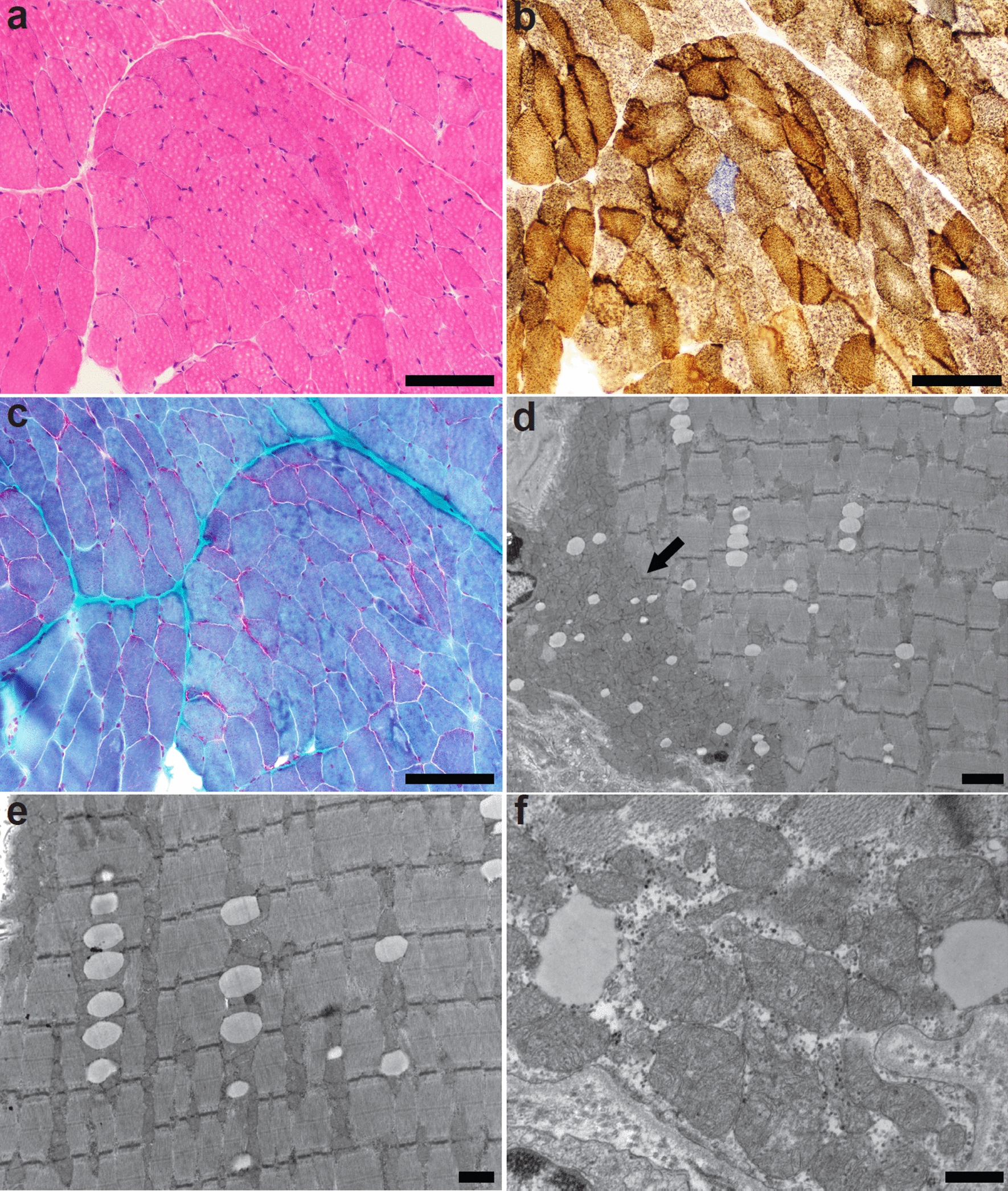
Histopathologic and ultrastructural findings of skeletal muscle biopsy. Antemortem skeletal muscle biopsy showed mild variation in muscle fiber diameter by light microscopy on H&E-stained sections **a** Dual staining for cytochrome C oxidase (COX) and succinate dehydrogenase (SDH) showed fibers with abnormal subsarcolemmal accumulations of mitochondria and a single COX negative fiber **b** Modified Gomori trichrome staining demonstrated the abnormal subsarcolemmal mitochondrial accumulation without evidence of ragged red fibers **c** Ultrastructural examination of myofibers showed aggregates of mitochondria in the subsarcolemmal space (black arrow) **d**, corresponding with the subsarcolemmal pads seen by light microscopy and within the sarcomeric apparatus **e**. Individual mitochondria exhibited ultrastructural abnormalities, including enlargement and formation of irregular cristae **f** Size bars are 100 μm (panels **a-c**), 2 μm (panel **d**), 1 μm (panel **e**), and 400 nm (panel **f**)

Antemortem muscle biopsy of the quadriceps was frozen and stained according to the typical clinical protocol at the University of Iowa histology laboratory, including stains for hematoxylin and eosin (H&E), modified Gomori trichrome, nicotinamide adenine dinucleotide (NADH), succinic dehydrogenase (SDH), a dual stain for SDH and cytochrome C oxidase (COX), and antibodies to the fast and slow myosin heavy chains. H&E stained sections showed skeletal muscle with mild variation in fiber size. The fibers were mildly hypotrophic, ranging in approximate diameter from 32 to 80 μm, with a median of 47 μm, measuring cross-sectional fibers along the shortest axis where appropriate. The fiber type distribution was within normal limits (Fig. 2a). A single COX-negative fiber was identified (Fig. 2b). There were numerous fibers with increased density of sarcoplasmic mitochondria and subsarcolemmal pads. Many of the subsarcolemmal pads were nearly circumferential, although there were no definite ragged-red fibers seen on modified Gomori trichrome stain (Fig. 2c). Ultrastructural examination by electron microscopy examination revealed regions of mitochondrial accumulation, including clusters of mitochondria throughout the sarcomeric apparatus and regions of subsarcolemmal accumulation (Fig. 2d, e). Many mitochondria were enlarged and contained electron-dense inclusions (Fig. 2f). Definite paracrystalline inclusions and concentric cristae were not identified. These findings were consistent with mitochondrial accumulation and ultrastructural mitochondrial cytopathy supportive of abnormal mitochondrial function.

A complete autopsy was performed, which included a specific focus on the brain and spinal cord. Peripheral nerve roots from the cauda equina were sampled, embedded in cross sections in epon, and stained with toluidine blue. No additional skeletal muscle was sampled at autopsy. The brain was fixed in 20% formalin for 15 days for neuropathologic examination. Gross examination of the brain at autopsy revealed a brain weight of 1400 g, with evidence of prior biopsy in the right frontal lobe. Coronal sections of the cerebral hemispheres revealed multiple confluent cystic lesions in the bilateral subcortical and periventricular white matter (Fig. 1b), involving the corpus callosum (Fig. 1d). The lesions extended from the bilateral anterior frontal lobes to the level of the basal ganglia and right mesial temporal lobe. The lesion in the right mesial temporal lobe demonstrated dusky discoloration and softening without distinct cavitation. All lesions spared the gray matter of the cerebral cortex, basal ganglia, thalamus, cerebellum, and brainstem. A confluent white-tan lesion was also identified in the bilateral posterior aspect of the spinal cord extending from the cervical cord to conus medullaris.

Extensive sectioning of the brain was performed, including sampling of the cavitary lesions, areas of dusky discoloration, and white-tan lesion in the spinal cord, as well as routine sectioning including watershed regions, hippocampus, and cerebellum. These sections were embedded in paraffin and processed in the usual fashion in the University of Iowa Department of Pathology histology laboratory. Sections of the lesion were stained with H&E, luxol fast blue (LFB) counterstained with H&E, and immunohistochemical stains for antibodies to neurofilament (2F11; 1:100), SOX10 (EP268; 1:400), CD45 (2B11 + PD7/26, 1:500), CD3 (polyclonal, 1:800), CD20 (L26, 1:400), CD163 (10D6, 1:800), and glial fibrillary acidic protein (GFAP) (GA-5, 1:400).

**Fig. 3:**
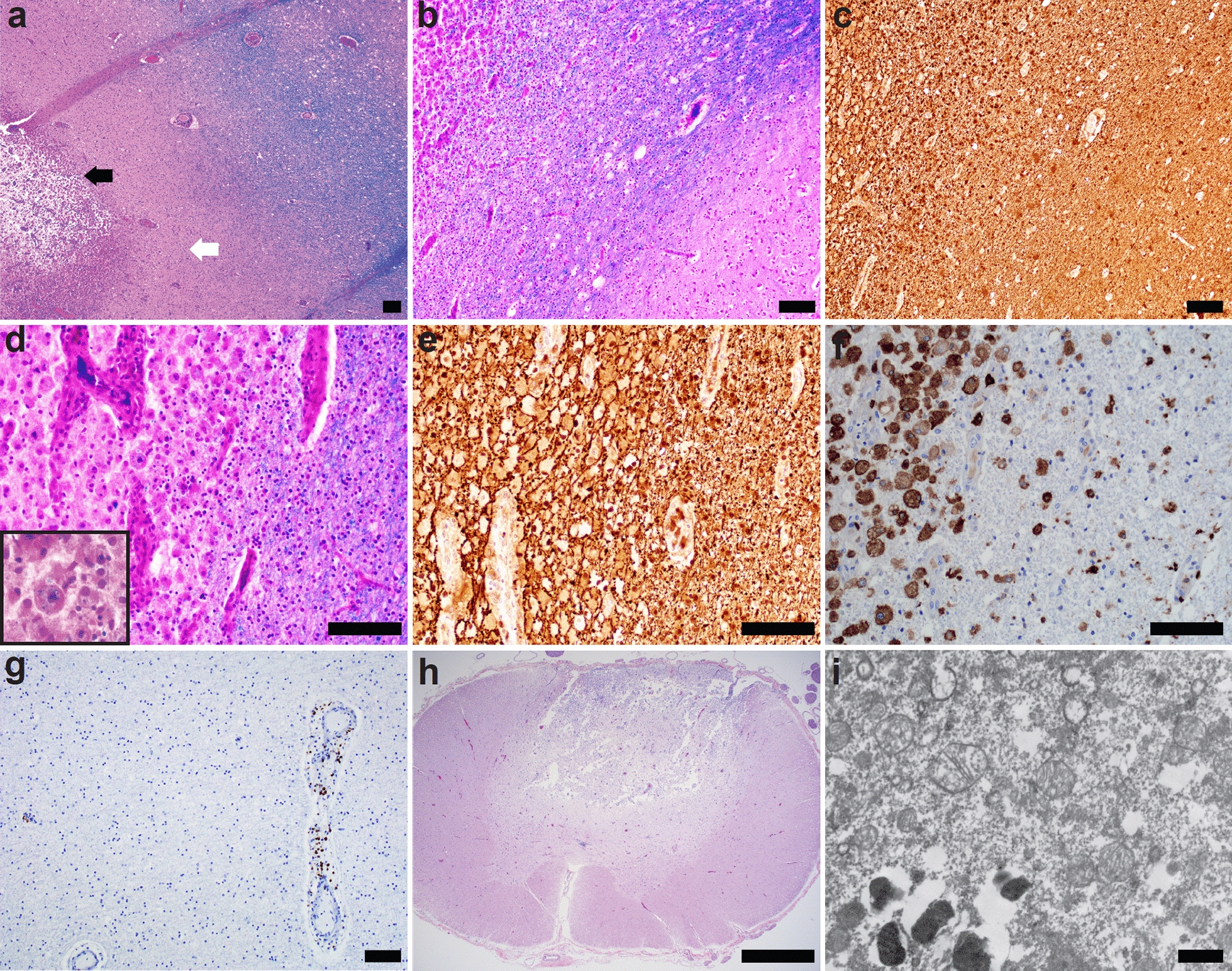
Histopathologic findings of brain at autopsy. Paraffin-embedded sections of the grossly solid lesions stained with Luxol fast blue / hematoxylin and eosin (LFB/H&E) demonstrated a central core of macrophage infiltration (black arrow), a rim of demyelinated axons (white arrow), and a clear demarcation from the myelinated white matter **a** LFB staining of the white matter adjacent to the cavitary lesions demonstrated conspicuous myelin preservation of the overlying subcortical U-fibers **b** and preservation of axons by neurofilament staining **c** In the areas of macrophage infiltration, there was evidence of myelin within macrophages on LFB (inset) **d**, preservation of axons by neurofilament staining **e**, and the macrophages were positive for CD163 **f**. Lymphocytic inflammation was scarce in the lesions, with only scattered parenchymal and perivascular CD3 + T-lymphocytes **g** H&E-stained sections of the spinal cord at all levels showed similar lesions with prominent involvement of the dorsal columns and sparing of corticospinal tracts **h** Ultrastructural analysis of cortical neurons did not identify definitive abnormal mitochondria (i). Size bars are 100 μm (panels **a-g**), 1 mm (panel **h**), and 600 nm (panel **i**)

Microscopic examination of the confluent white matter lesions showed variable stages of progression from early demyelination to complete cavitation. The most solid lesion – the non-cavitary, discolored region in the right mesial temporal lobe – demonstrated areas of transition from macrophage-rich infiltrate to preserved myelinated white matter with an intervening region of demyelinated axons (Fig. 3a). This region also showed greater preservation of axonal integrity and a less significant degree of axonal injury. The subcortical U-fibers were conspicuously spared from this demyelination on LFB staining (Fig. 3b) with consistent labeling of axons by neurofilament staining in this region and in the underlying lesional white matter (Fig. 3c). The most cavitary regions demonstrated only scattered macrophages and complete loss of white matter, with negative neurofilament and LFB staining in these areas. The periphery of the cavitary lesions were macrophage rich, with prominent neovascularization, myelin loss, and evidence of myelin within macrophages (Fig. 3d). Notably, both the periphery of the cavitary lesions and the discolored temporal lesion contained a range of axonal integrity, from largely preserved axons (Fig. 3e) to swollen, damaged axons with axonal spheroids on neurofilament staining. There were only occasional foci of perivascular lymphocytic cuffing. Immunohistochemical staining for CD3, CD20, CD45, and CD163 confirmed the extensive macrophage infiltrate (Fig. 3f), and occasional CD3 + perivascular T-lymphocytes (Fig. 3g), but demonstrated the absence of a significant parenchymal inflammatory component. Sections of the spinal cord revealed similar findings to the subcortical white matter lesions, with macrophage infiltrate in the dorsal columns, but with relative preservation of the anterior horns and anterior and lateral corticospinal tracts (Fig. 3h). Ultrastructural evaluation of the cerebral cortex was performed retrospectively using tissue previously embedded in paraffin. Where best visualized within neuronal cytoplasm, mitochondria did not show definite mitochondrial abnormalities on limited examination (Fig. 3i).

Sampled peripheral nerve roots from the cauda equina showed a normal population of large and small myelinated axons. There was no evidence of axonal degeneration, axonal loss, inflammation, thinly myelinated axons, or onion bulbs.

## Discussion and conclusions

We report the skeletal muscle and CNS neuropathologic findings in a patient with likely deficiency in complex I of the mitochondrial respiratory chain in association with compound heterozygous likely pathogenic variants in *NDUFV1.* Of the two variants identified in our patient, one (c.365C > T, p.P122L) has been previously reported [2, 7, 14]. The histopathology of the muscle and brain have not been previously described for this variant, but previous reports of biochemical analysis of skeletal muscle have demonstrated complex I deficiency. The other variant (c.565 T > C, p.S189P) has not been previously reported. This missense variant has been reported at an allele frequency of < 0.002% (gnomAD) in the general population and in silico analysis supports a deleterious effect on the protein structure. Parental testing demonstrated that this variant was in trans with the known pathogenic variant. The pathologic findings support the conclusion that these compound heterozygous variants cause a deficiency in complex I and subsequent progressive cavitating leukoencephalopathy. Therefore, these findings also provide evidence of the pathogenicity of the c.565 T > C, p.S189P variant.

The term progressive cavitating leukoencephalopathy was first described in 2005 by Naidu et al. in a series of young patients with distinct clinical and radiologic findings but unknown genetic alterations [10]. The authors proposed an autosomal recessive inheritance pattern and suggested a mitochondrial origin based on occasional elevations of lactate in the CSF and serum [10]. The connection between these cystic changes on MRI and alterations in the gene *NDUFS1,* which encodes for a mitochondrial subunit in complex I, was later described in 2011 [5]. Additional reports have described these imaging findings in association with *NDUFS1* and other nuclear genes encoding mitochondrial subunits including *NDUFV1* and *NDUFV2* [2, 5, 8, 11, 13, 14]. Most of the patients described in these cases presented in infancy, an age range of 6–14 months, with regression of motor milestones, spasticity, and hypotonia, which often stabilized after the acute presentation [2, 5, 8, 11, 13, 14]. It is not clear in our patient if she had an acute presentation in early childhood, but by 4 years of age, she was noted to have gait abnormalities that were stable until her acute presentation in her early twenties. Previous reports described presentation during childhood, and only one case described two patients who were in their teens [2]. There are no published reports of any patients with *NDUFV1* variants living into their twenties before they had significant clinical presentation and decline, as with the patient we describe.

The comprehensive neuropathologic findings of progressive cavitating leukoencephalopathy associated with pathogenic variants in *NDUFV1* have not been previously described. The histologic findings at autopsy demonstrate a temporal heterogeneity from early demyelination to cavitation. The conclusion of early demyelination is supported by solid lesions showing evidence of myelin loss, relatively preserved neurofilament staining, and dense macrophage infiltrate with myelin inclusions in macrophage cytoplasm. Other pathologic considerations were excluded by histopathologic evaluation. The degree of axonal preservation was not consistent with subacute infarct, and there was not significant evidence of ischemic injury or necrosis. The white-matter distribution, sparing cortical areas and deep gray nuclei, excluded other described mitochondrial pathologies such as MELAS or Leigh syndrome. Finally, no pathologic features of any other well-characterized leukodystrophy, such as a lysosomal storage disorder, were seen.

These pathologic findings are consistent with and expand upon previous descriptions of patients with complex I deficiency. The pathology in the brain and spinal cord showed many similarities to the pathologic findings in the autopsies described by Naidu et al. [10] (with no genetic correlates published at that time) and the biopsy described by Bindu et al. [1] (with an *NDUFA1* variant) [1, 10]. Our findings were also present in our patient’s antemortem brain biopsy, but the limited sample size made it challenging to correlate with the clinical picture. The full nature of the lesions could not be appreciated until autopsy examination. The white matter involvement with sparing of deep gray nuclei is consistent with the described imaging findings in previous reports [2, 5, 8, 10, 11, 13, 14]. There was preservation of the subcortical U-fibers, which was also described by Naidu and colleagues [10]. Finally, involvement of the dorsal columns in the spinal cord was described by Naidu and colleagues, and this finding has been noted radiographically in other patients with *NDUFV1* variants [3].

The skeletal muscle findings are consistent with complex I deficiency as the underlying etiology for the patient’s presentation. Case series evaluating complex I deficiency have described a decrease in enzyme activity of complex I, but the histomorphology is either described as normal or not described at all [2, 5, 8]. In our case, the histomorphology by light microscopy was subtle, but there was subsarcolemmal accumulation of mitochondria in a subset of myofibers, and a rare COX-negative fiber was identified. The abnormalities in mitochondrial morphology were strongly supported by ultrastructural analysis. The ultrastructural findings are themselves not entirely specific. Together with the genetic findings, however, they provide evidence of mitochondrial dysfunction, consistent with a complex I deficiency.

Lastly, this case provides additional evidence that mitochondrial dysfunction may result in demyelinating pathology. Bindu et al. [1] suggested that mitochondrial leukoencephalopthies represent a “border zone” between inherited and acquired demyelination and that mitochondrial leukoencephalopathies may mimic acquired demyelination [1]. Variants in genes coding for complex I have been described in association with multiple sclerosis, and complex I deficiencies have been identified in patients with multiple sclerosis [6, 12]. Finally, recent studies examining the pathophysiology of multiple sclerosis have identified mitochondrial dysfunction and related oxidative stress to the oligodendrocytes as a component of the acute demyelinating process [9]. Interestingly, sparing of the subcortical U-fibers is also seen in other white-matter disease processes, such as vanishing white matter disease and leukodystrophies due to other metabolic aberrations, but is generally not seen in acquired inflammatory processes such as multiple sclerosis or acute disseminated encephalomyelitis (ADEM). This finding suggests the underlying process in this mitochondrial leukoencephalopathy is one of myelin metabolism in oligodendrocytes rather than direct injury to oligodendrocytes. Based on the pathologic findings in our case, we hypothesize that mitochondrial dysfunction caused by complex I deficiency is an inciting event in the oligodendrocytes, resulting in the demyelinating process and ultimate cavitation.

Our report has several important limitations. First, while the overall pathology was pauci-inflammatory, the patient received extensive immunomodulatory treatment. Therefore, the extent to which the underlying disease process is inflammatory in nature is difficult to discern. Second, ultrastructural examination of cortical tissue did not establish evidence of mitochondrial abnormalities or mitochondrial accumulation, best observed in the cytoplasm of neuronal cell bodies. Notably, this evaluation was performed retrospectively on paraffin-embedded tissue, limiting assessment of mitochondrial morphology and distribution. Despite this finding, the strong evidence of pathogenicity of the *NDUDFV1* variants and mitochondrial abnormalities in skeletal muscle together support the mitochondrial etiology of this disease process.

This case highlights the pathologic features present in progressive cavitating leukoencephalopathy due to pathogenic variants in *NDUFV1,* including a variant not previously described as pathogenic, *NDUFV1* (c.565 T > C, p.S189P). We demonstrate an active demyelinating process with progressive destruction of brain parenchyma and ultimate cavitation. This is coupled with mild histopathologic abnormalities and more pronounced ultrastructural mitochondrial abnormalities in the skeletal muscle biopsy. While no definite ultrastructural mitochondrial abnormalities were observed in cortical tissue, the remaining findings and genetic results provide evidence that the neurodegenerative process is related to mitochondrial dysfunction due to complex I deficiency. Finally, this case offers an example of how mitochondrial dysfunction may result in demyelinating pathology.

## Data Availability

Data sharing is not applicable to this article as no datasets were generated or analyzed during the current study.
